# Editorial: Novel insights connecting telomere biology to cancer development and progression

**DOI:** 10.3389/fonc.2024.1405618

**Published:** 2024-04-16

**Authors:** Ekta Khattar, Erica Salvati

**Affiliations:** ^1^ Sunandan Divatia School of Science, SVKM’s NMIMS (Deemed to be) University, Mumbai, India; ^2^ Institute of Molecular Biology and Pathology, National Research Council, Rome, Italy

**Keywords:** telomere, telomere length, telomerase, cancer, shelterin complex, alternative lengthening of telomeres

## Introduction

Telomere length maintenance represents a unique connection between replicative senescence and carcinogenesis, functioning like a double-edged sword. Telomere shortening suppresses cancer development in normal cells, limiting their replicative potential. Contrarily, in precancerous lesions, telomeric dysfunction, arising from telomere shortening, may promote genomic instability, fueling cancer development. Therefore, investigating proteins and genes involved in telomere homeostasis offers great opportunities for developing targeted therapies and for identifying novel markers of cancer initiation and progression.

More than 80% of cancers attain replicative immortality predominantly through activation of telomerase, which mostly occurs through TERT re-expression during cancer initiation and development (1, 2). Telomerase enzyme consists of a catalytic subunit called telomerase reverse transcriptase (TERT), which adds nucleotides to the extending 3’ telomeric DNA strand by using an RNA template called telomerase RNA component (*TERC or TR*) (3). The presence of functionally active telomerase confers cells with the ability to undergo infinite cell division without losing essential genetic elements. 10 to 15% of cancer cells elongate telomeres using alternative lengthening of telomeres (ALT) using telomeric recombination (4).

Telomere metabolism involves specialized functional complexes located at telomeres like shelterin and CST complexes. Shelterin is a six-member complex with a high affinity for telomeric DNA. It comprises TRF1, TRF2, POT1, TPP1, TIN2 and RAP1 proteins. Shelterin complex is mainly involved in the inhibition of inappropriate activation of DDR at telomeres, which could promote telomere recombination and genome instability (5). CST is a trimeric complex consisting of CTC, Stn1, and Ten1 in humans (6). CST complex associates with single-stranded telomeric overhang and prevents telomerase association with telomeres while promoting the association of DNA polymerase α/primase to promote telomeric C-strand fill-in reaction (7–9). This coordination of G and C-strand synthesis occurs in the late S-early G2 phase when telomerase extension of telomeric G-strand is completed (10, 11).

This editorial summarizes the recent achievements in the field of telomere biology and cancer with a collection of two case reports, original research articles and brief research reports, and a review article published over a few months in the Frontiers Research Topic *Novel insights connecting telomere biology to cancer development and progression*.

In a case report, He et al. reported a mutation in the CST component gene, CTC1, which was associated with a benign form of cancer, namely liver hemangioma. Hemangiomas are generally asymptomatic but can be complicated if they become giant and diffuse, where they tend to be extrahepatic as well. Authors performed whole exome sequencing and identified heterozygous variants of the *CTC1* gene: a splicing site mutation, c.435 + 9A>C, and a missense mutation, c.3074C>T (p.Ala1025Val), which may be involved in vascular disease pathology.

In the second case report of this Research Topic, Nisar et al. report a pathological mutation in the RTEL1 gene (regulator of telomere elongation helicase 1) in a dyskeratosis congenital (DKC) patient. Authors performed targeted sequencing of nine genes involved in DKC pathogenesis, shortlisted based on literature. They report a novel homozygous RTEL1 gene variant c.2060C>T (p.Ala687Val) in the patient. Functional assay of the mutant protein is not reported.

The onset of telomere lengthening mechanisms is an early event in cancer development and the comprehension of this process is a major goal for cancer research. In an original research article, Udroiu et al. investigated the effects of the inhibition of ATRX and p53 proteins in aged primary fibroblasts on ALT activity. Udroiu et al. reported the activation of the ALT pathway upon p53 silencing in aged fibroblasts, associated with an increased telomeric recombination due to a de-repression of the homologous recombination (HR) pathway. Since ALT cancers are characterized by p53 and ATRX mutations, this paper highlights a potential mechanism of induction of ALT activity occurring in aged human tissues during oncogenesis.

A novel regulatory mechanism, acting at ALT-positive telomeres, is reported by Tang et al. In this research article, Tang and coauthors present experimental evidence that the shelterin protein TRF2 recruits MRE11 and UBR5 to suppress the RNF168-53BP1 dependent cNHEJ mediated telomeric fusion at ALT telomeres. Of note, loss of this regulation would be tumor suppressive. This finding could pave the way for the generation of small molecules or peptides that could specifically disrupt this mechanism triggering telomeric shortening and cancer cell death.

Telomere length is a hallmark for cancer cells, and has been frequently correlated with cancer aggressiveness, response to treatment or disease recurrence. Two research articles in this Research Topic addresses this important point.

Li et al. investigated the correlation between telomere length and prognosis of breast cancer with varying expression of estrogen receptor (ER) using the Mendelian randomization approach. The authors report that longer telomeres could predict a poor prognosis of ER-negative breast cancer, while no significant correlation was observed between ER-positive breast cancer and telomere length. This substantially confirms the telomere lengthening as a biomarker for cancer prognosis and as a target for cancer treatment.

An important role of telomere length in prediction of the risk of post-transplant malignancy (PTM) is reported by Petrara et al. Telomere erosion in activated and exhausted T and B cells at baseline determined a higher risk of PTM in liver-transplanted patients for hepatocellular cancer development compared to that receiving transplant due to other reasons. This suggests that telomere attrition, determined by immune activation and exhaustion, may be useful to predict the risk of PTM occurrence. In addition, this finding suggests that T-cell senescence can represent an additional risk factor for tumor onset in LT-HCC.

Targeting telomere lengthening and telomeric components using direct inhibitors and their combination with other effective anti-tumor drugs represents a promising therapeutic opportunity to selectively hit cancer cells.

In a research article, Chebly et al. investigated the effect of epidrugs on the proliferation of Sézary cells using *in vitro* studies. Sézary syndrome (SS) is an aggressive leukemic variant of cutaneous T-cell lymphomas in which the TERT gene is re-expressed. The group of Chebly et al. report that 5-azacytidine, romidepsin, and vorinostat epidrugs combination could downregulate TERT expression and reduce tumorigenic potential of SS cells.

Finally, in a review article, Banikazemi et al. reviewed in detail the most recent achievements obtained with the employment of coumarins derivatives for the treatment of gastrointestinal cancer (GC). Banikazemi et al. analysed in detail the mechanistic action of the natural product coumarin for its anti-tumor activity. As reported, coumarins exhibit a variety of functions, including hormone antagonism, DNA alkylation, angiogenesis inhibition, telomerase, topoisomerase and carbonic anhydrase inhibition, apoptosis inhibition, antimitotic effect etc, and still represent a valid therapeutic opportunity for the treatment of GC.

Overall, the Research Topic strengthen the importance of telomere maintenance in the development and progression of cancer and its therapeutic potential as target and as biomarker.

Nevertheless, many of the studies are specific to tumor types and disease conditions and require further investigation with strong experimentation and validation. Further studies in the field will help to develop a better understanding of the role of telomere-associated proteins and genes in the initiation, development, and treatment of various cancer types.

## Author contributions

EK: Writing – review & editing, Writing – original draft. ES: Writing – review & editing, Writing – original draft.
